# Metastasis-Directed Radiotherapy for Oligometastatic Castration-Resistant Prostate Cancer

**DOI:** 10.7759/cureus.13199

**Published:** 2021-02-07

**Authors:** Atsuto Katano, Hideomi Yamashita, Keiichi Nakagawa

**Affiliations:** 1 Radiology, The University of Tokyo Hospital, Tokyo, JPN

**Keywords:** radiotherapy (rt), metastasis-directed therapy, recurrence, castration-resistant prostate cancer, oligometastasis

## Abstract

The treatment effects of metastasis-directed therapy in patients with oligometastatic disease have received much attention. In our case, a 72-year-old man with oligometastatic castration-resistant prostate cancer was referred to our hospital. The patient had undergone radical radiotherapy with a total dose of 76 Gy in 36 fractions for localized prostate cancer nine years prior to the first visit. Positron emission tomography showed a slight increase in accumulation in the para-aortic lymph nodes. The patient received conventional radiotherapy at a total dose of 50 Gy in 25 fractions to the para-aortic region as oligometastasis-directed local therapy. After radiotherapy, his prostate-specific antigen (PSA) level decreased slightly, but it increased again soon after. According to the results of positron emission tomography, the accumulation around the para-aortic lymph nodes had decreased; however, a slight increase in accumulation in the sub/supra-clavicular lymph nodes was observed. He received radiotherapy at a total dose of 50 Gy in 25 fractions to the sub/supra-clavicular region. We confirmed a significant reduction in lesion volume and a downward trend in PSA levels.

Metastasis-directed therapy has shown remarkable effectiveness in controlling disease without severe treatment-related adverse events. Metastasis-directed therapy is considered as one of the treatment options in patients who need salvage therapy.

## Introduction

Oligometastasis is a type of metastasis in which only a limited number of metastases are identified in patients with cancer. The concept was first proposed by Hellman and Weichselbaum in 1995 [1]. While the standard treatment for patients with distant metastases in many types of cancer is systemic therapy, oligometastasis can be treated with radical local therapy, including surgery and radiotherapy, to cure the disease. The efficacy of local therapy for liver metastasis from colorectal cancer has long been known. Surgical resection of liver metastasis is an effective treatment for selected colorectal cancer patients. This approach was reported to achieve five-year survival rates of approximately 40% [2].

In recent years, evidence regarding the treatment outcomes of oligometastasis-directed therapy has been accumulating. Stereotactic ablative radiotherapy for comprehensive treatment of oligometastatic cancers (called SABR-COMET trial), an international randomized phase II trial, has suggested useful results for patients with oligometastasis [3]. Ninety-nine patients with well-controlled primary tumors and oligometastatic lesions were randomized in a 1:2 ratio between the palliative care arm and the stereotactic ablative radiotherapy (SABR) arm. The SABR arm was shown to significantly prolong overall survival. The five-year overall survival rate in the palliative care arm was 17.7%, while that in the SABR arm was 42.3%. Another multi-institutional, phase II randomized study reported the advantage of metastatic local therapy in patients with stage IV non-small-cell lung cancer with three or fewer metastases after front-line systemic therapy [4]. The median progression-free survival improved from 5.6 months in the maintenance therapy or observation group to 14.2 months in the local therapy group. Moreover, the median overall survival also improved from 17.0 months to 41.2 months, without severe treatment-related toxicities. This trial was closed early due to the significant efficacy benefit observed in the metastatic local therapy arm.

Further evidence is needed regarding oligometastatic treatment focusing on specific primary cancer types. Herein, we present the case of a patient with oligometastatic castration-resistant prostate cancer treated with metastasis-directed radiotherapy.

## Case presentation

A 72-year-old man was referred to our hospital due to an elevated prostate-specific antigen (PSA) level during androgen deprivation therapy (ADT). Nine years prior to admission, he had undergone radiotherapy with a total dose of 76 Gy in 36 fractions for localized prostate cancer (PSA level, 25.55 ng/mL; Gleason score = 5+4; and cT1cN0M0 stage). The treatment was successful, and his PSA level decreased to 0.02. However, two years after treatment, he experienced biochemical failure, and his PSA level increased. Therefore, he was started on ADT comprising bicalutamide and leuprorelin. Subsequently, his PSA level dropped to 0.024 ng/mL a year prior to the first visit of our department and then rose again to 2.5 ng/mL; thus, he was referred to our department. His serum testosterone level was found to be 38.8 ng/dL, and he was diagnosed with castration-resistant prostate cancer (CRPC).

**Figure 1:**
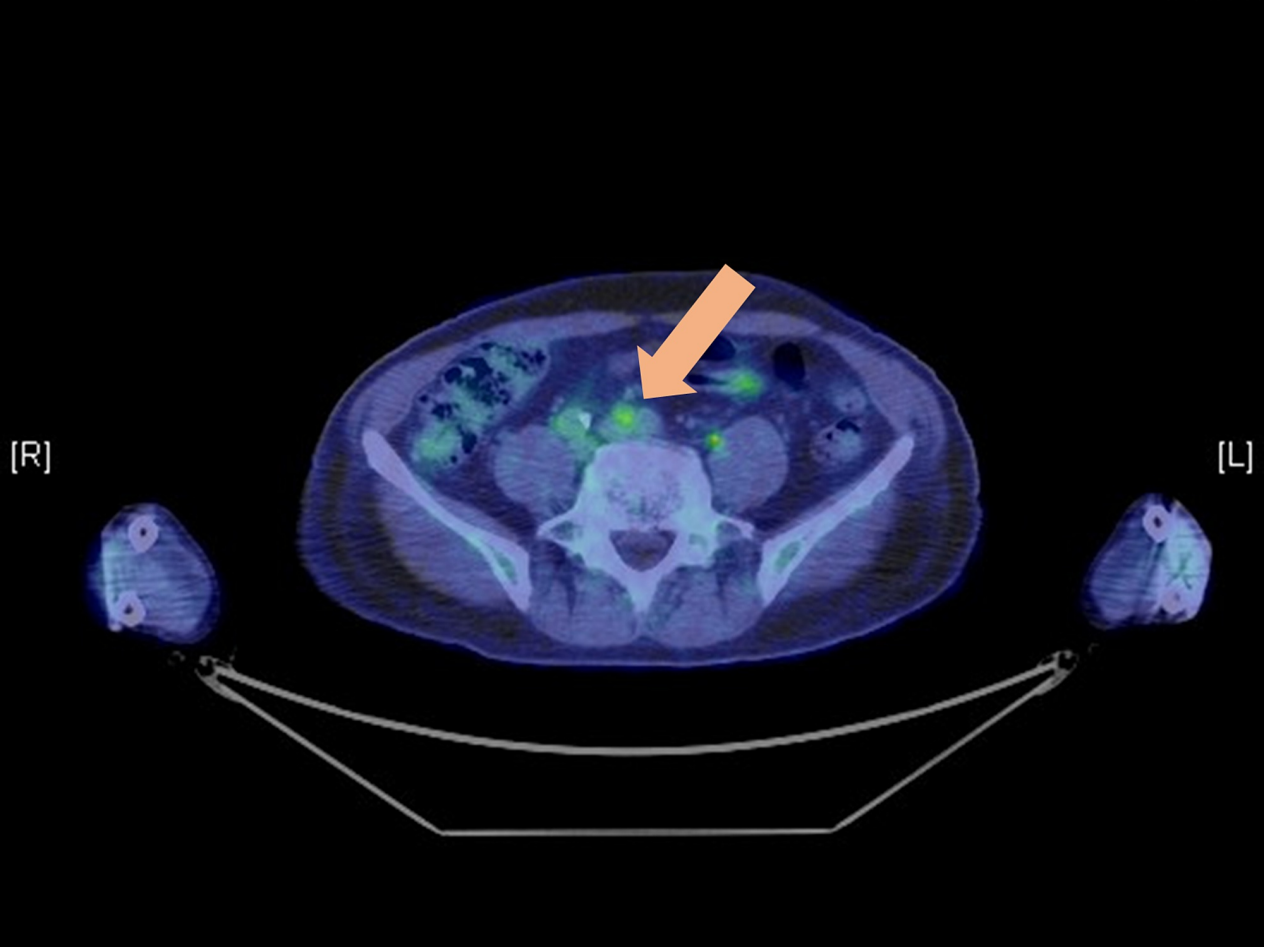
Positron emission tomography combined with computed tomography Positron emission tomography combined with computed tomography showing slightly higher fluorodeoxyglucose uptake in the para-aortic lymph nodes

Positron emission tomography (PET) showed a slight increase in accumulation in the para-aortic lymph nodes (fluorodeoxyglucose maximum standardized uptake value [SUV max]: 3.1) (Figure 1).

**Figure 2:**
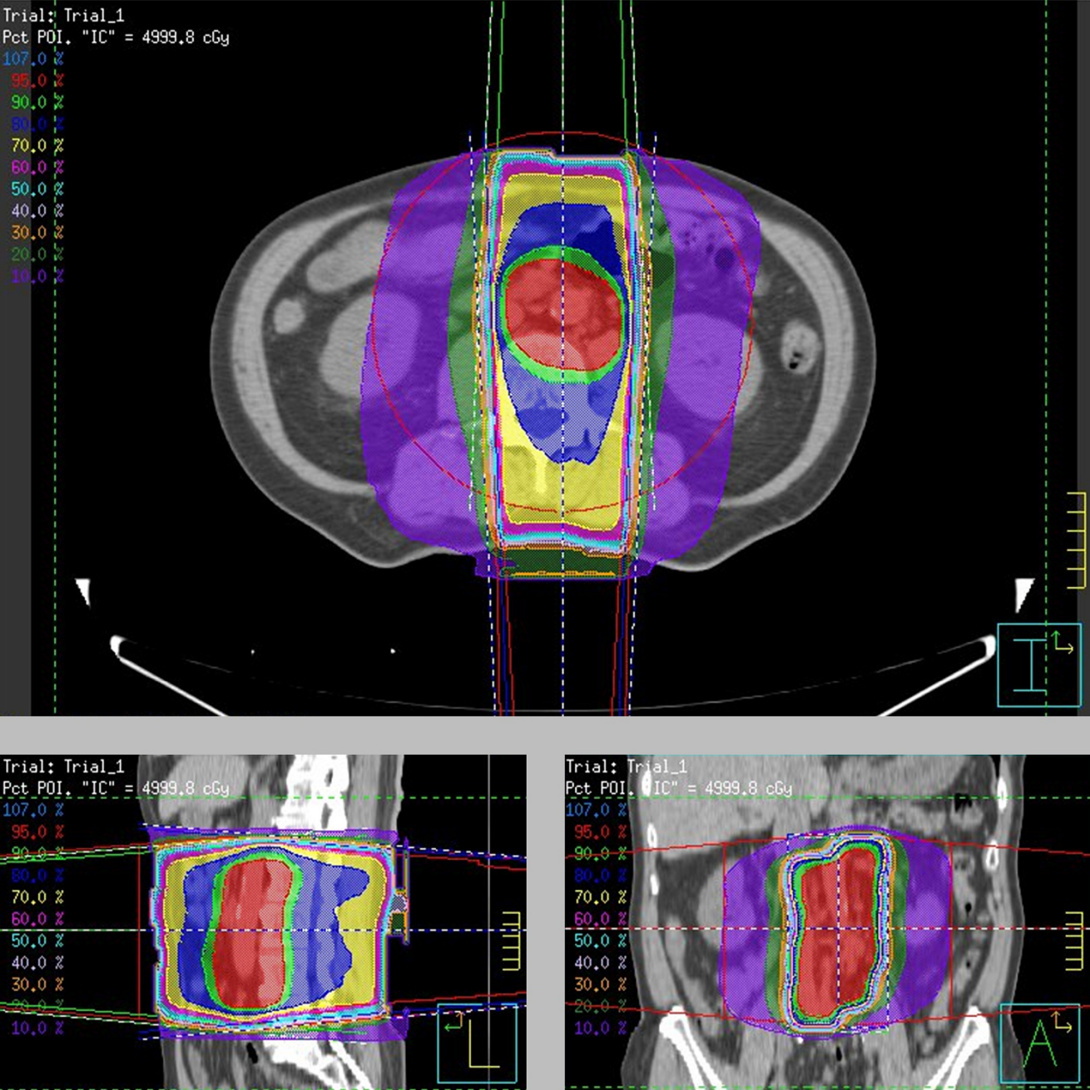
Radiotherapy planning for the para-aortic lymph nodes Distribution of the conventional radiotherapy dose for the para-aortic lymph nodes. The isodose lines are shown in the upper left corner. The 95% isodose of the prescribed dose is indicated as the red-colored area.

In our case, SABR was not suitable due to extensive para-aortic lymph node enlargement. We decided to administer conventional radiotherapy at a total dose of 50 Gy in 25 fractions to the para-aortic lymph nodes as metastasis-directed therapy (Figure 2).

**Figure 3:**
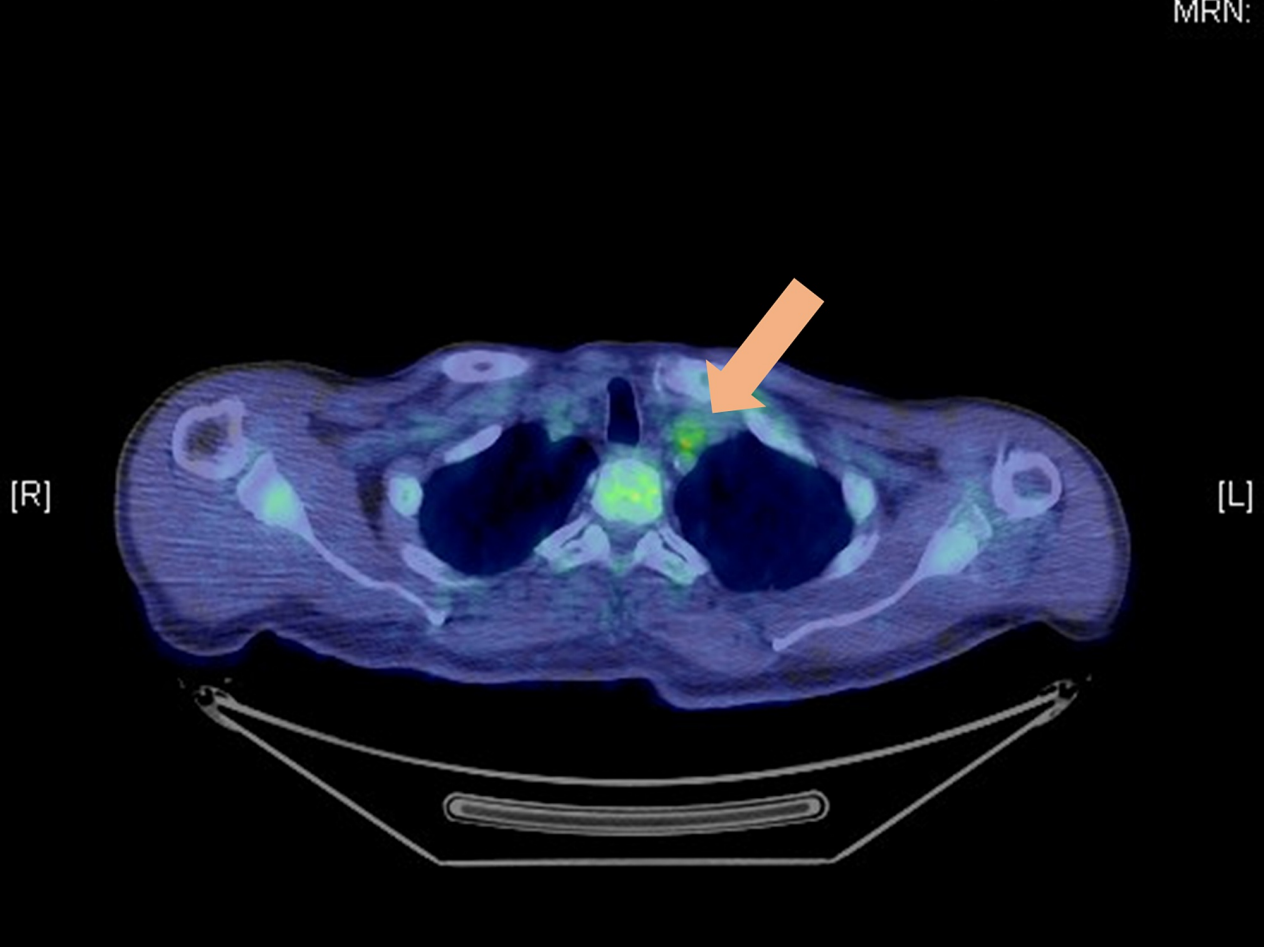
Positron emission tomography combined with computed tomography Positron emission tomography combined with computed tomography showing slightly higher fluorodeoxyglucose uptake in the sub/supra-clavicular lymph nodes

Only grade 1 nausea developed as a radiation-related adverse event. No hematological adverse events were observed. After radiotherapy, his PSA level decreased slightly, but it increased again soon after. On PET, the accumulation around the para-aortic lymph nodes was obscured; however, there was a slight increase in accumulation in the sub/supra-clavicular lymph nodes (SUV max: 3.0-3.3) (Figure 3).

**Figure 4:**
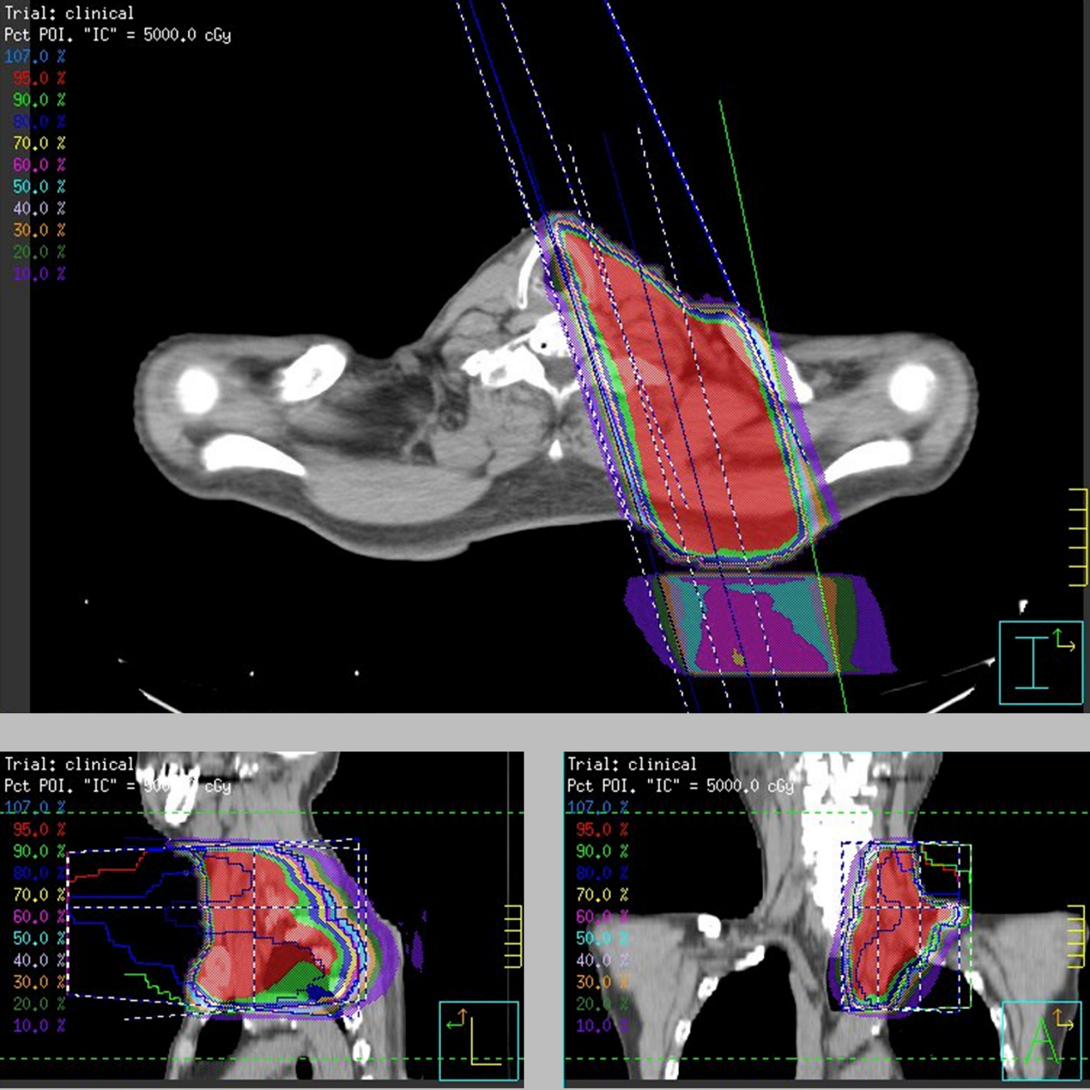
Radiotherapy planning for the sub/supra-clavicular lymph nodes Distribution of the conventional radiotherapy dose for the sub/supra-clavicular lymph nodes. The isodose lines are shown in the upper left corner. The 95% isodose of the prescribed dose is indicated as the red-colored area.

The patient received radiotherapy at a total dose of 50 Gy in 25 fractions to the sub/supra-clavicular region (Figure 4).

**Figure 5:**
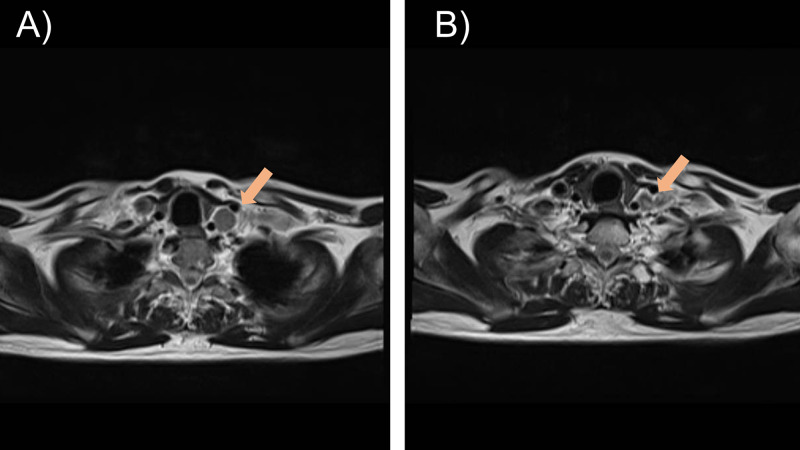
Magnetic resonance imaging Magnetic resonance imaging showing a significant reduction in the size of the sub/supra-clavicular lymph nodes A) before radiotherapy and B) after radiotherapy

**Figure 6:**
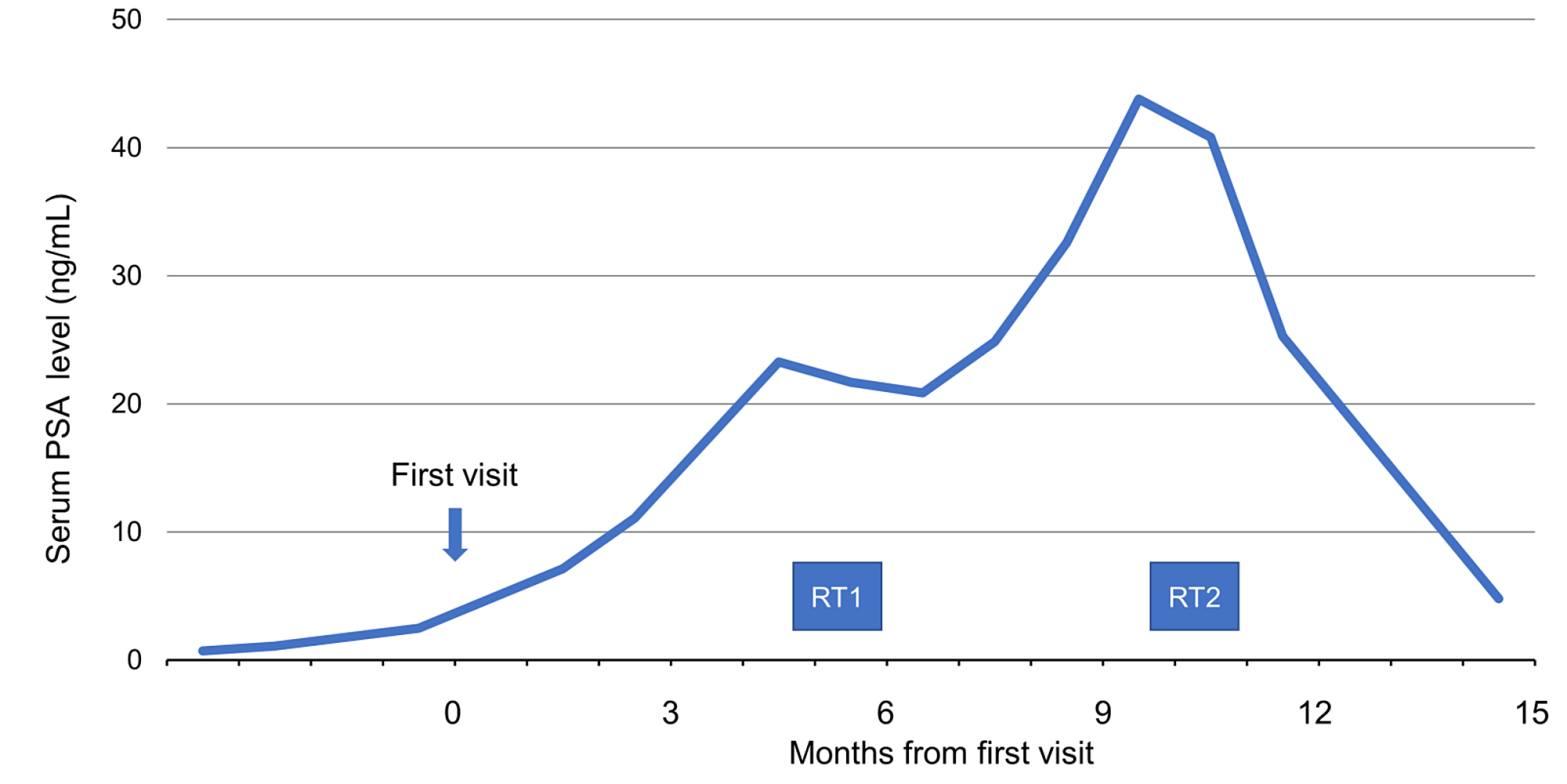
Trend of prostate-specific antigen (PSA) levels Trend of prostate-specific antigen (PSA) levels from the first visit to our institution. After the first radiotherapy (RT1), the PSA level decreases slightly but increases soon after. After the second radiotherapy (RT2), the serum PSA level shows a downward trend.

Only grade 1 radiation dermatitis developed as an adverse event, and he had no hematological adverse events. We then confirmed volume reduction of the lesion by magnetic resonance imaging (Figure 5) and a downward trend in the PSA level (Figure 6). No significant abnormal accumulation was found in whole-body PET images after five months of the radiotherapy.

## Discussion

Many patients with recurring or advanced prostate cancer initially respond to ADT, and most patients show progression to CRPC after a few years [5]. Systemic treatment options for CRPC are expanding, including new androgen-targeted agents such as enzalutamide, apalutamide, darolutamide, and abiraterone and a next-generation taxane, cabazitaxel [6].

The efficacy of metastasis-directed therapy has been revealed for oligometastatic hormone-sensitive prostate cancer. In a phase II randomized clinical trial of observation versus stereotactic ablative radiation for oligometastatic prostate cancer, known as the ORIOLE trial, the patients were randomized in a 2:1 ratio to either the SABR treatment or surveillance group [7]. A total of 54 study participants were included: seven of 36 patients (19%) in the SABR group showed disease progression within six months, compared to 11 of 18 (61%) in the surveillance group. Another randomized phase II trial assessed the benefit of metastasis-directed therapy in terms of the initiation of ADT in 62 patients with oligorecurrent prostate cancer. The median ADT-free survival was 21 months in the ADT arm and 13 months in the surveillance arm [8]. Regarding oligo-progressive CRPC, a recent retrospective analysis reported that metastasis-directed therapy prolonged the time to PSA failure compared to systemic therapy alone [9].

There is no evidence-based standardized sequential therapy for CRPC. It is important to determine individual treatment methods based on an understanding of each patient’s characteristics, such as performance status, disease symptoms, presence of organ metastases, and the effect of prior therapy. Precision medicine, which is the practice of applying the most appropriate treatment method to individual patient information, might help this decision.

For example, in CRPC patients with gene mutations in the homologous recombination repair pathway, the poly (adenosine diphosphate‐ribose)‐polymerase‐1 inhibitor olaparib significantly prolonged progression-free survival compared to enzalutamide or abiraterone plus prednisone [10]. The hazard ratio for disease progression or death was 0.34 (95% confidence interval: 0.25-0.47, p < 0.001) in the olaparib arm. Moreover, the overall survival rate was also higher in the olaparib arm at the time of analysis, although this difference was not statistically significant.

Patients with oligometastatic prostate cancer include those who are clinically inhomogeneous. Evaluating and treating patients with oligometastatic prostate cancer is extremely difficult. Owing to the lack of knowledge regarding disease progression from the oligometastatic state to an extensive metastatic state, treatment decisions should be made carefully.

## Conclusions

We report a case of efficacious treatment of oligometastatic CRPC with metastasis-directed radiotherapy. We were able to suppress disease progression by conventional radiotherapy. Metastasis-directed local treatment was considered to be a reasonable treatment option without severe treatment-related toxicity.
